# Omega-3- and Resveratrol-Loaded Lipid Nanosystems for Potential Use as Topical Formulations in Autoimmune, Inflammatory, and Cancerous Skin Diseases

**DOI:** 10.3390/pharmaceutics13081202

**Published:** 2021-08-04

**Authors:** Ana R. Caldas, José Catita, Raul Machado, Artur Ribeiro, Fátima Cerqueira, Bruno Horta, Rui Medeiros, Marlene Lúcio, Carla M. Lopes

**Affiliations:** 1 CF-UM-UP, Centro de Física das Universidades do Minho e Porto, Departamento de Física da Universidade do Minho, 4710-057 Braga, Portugal; anaritapc1@gmail.com; 2 FP-I3ID, Fernando Pessoa Energy, Environment, and Health Research Unit/Biomedical Research Center (FP-ENAS/CEBIMED), Portugal and Faculty of Health Sciences, Fernando Pessoa University, 4200-150 Porto, Portugal; jcatita@ufp.edu.pt (J.C.); fatimaf@ufp.edu.pt (F.C.); ruimedei@ipoporto.min-saude.pt (R.M.); 3 Paralab, SA, 4420-392 Valbom, Portugal; 4 CBMA, Centro de Biologia Molecular e Ambiental, Departamento de Biologia, Universidade do Minho, 4710-057 Braga, Portugal; raulmachado@bio.uminho.pt; 5 IB-S (Institute of Science and Innovation for Bio-Sustainability), University of Minho, Campus de Gualtar, 4710-057 Braga, Portugal; 6 CEB, Centro de Engenharia Biológica, Universidade do Minho, 4710-057 Braga, Portugal; arturibeiro@ceb.uminho.pt; 7 CIIMAR/CIMAR, Interdisciplinary Centre of Marine and Environmental Research, 4450-208 Matosinhos, Portugal; 8 Molecular Oncology and Viral Pathology Group, Research Center of IPO Porto (CI-IPOP)/RISE@CI-IPOP (Health Research Network), Portuguese Oncology Institute of Porto (IPO Porto)/Porto Comprehensive Cancer Center (Porto.CCC), 4200-072 Porto, Portugal; bhorta@porto.ucp.pt; 9 CBQF—Centre for Biotechnology and Fine Chemistry, Associated Laboratory, Higher School of Biotechnology, Portuguese Catholic University, 4169-005 Porto, Portugal; 10 ICBAS—Instituto de Ciências Biomédicas Abel Salazar, Universidade do Porto, 4050-313 Porto, Portugal

**Keywords:** resveratrol, omega 3, topical skin administration, lipid nanosystems, COX inhibitors, NO inhibitory effect, antioxidant

## Abstract

Resveratrol (RSV) and omega 3 (ω_3_), because of their biological favorable properties, have become subjects of interest for researchers in dermocosmetic and pharmaceutical industries; however, these bioactives present technological limitations that hinder their effective delivery to the target skin layer. To overcome the stability and skin permeation limitations of free bioactives, this work proposes a combined strategy involving two different lipid nanosystems (liposomes and lipid nanoparticles) that include ω_3_ in their lipid matrix. Additionaly, RSV is only encapsulated in liposomes that provid an adequate amphiphilic environment. Each formulation is thoroughly characterized regarding their physical–chemical properties. Subsequently, the therapeutic performance of the lipid nanosystems is evaluated based on their protective roles against lipid peroxidation, as well as inhibition of cicloxygenase (COX) and nitric oxid (NO) production in the RWA264.7 cell line. Finally, the lipid nanosystems are incorporated in hydrogel to allow their topical administration, then rheology, occlusion, and RSV release–diffusion assays are performed. Lipid nanoparticles provide occlusive effects at the skin surface. Liposomes provide sustained RSV release and their flexibility conferred by edge activator components enhances RSV diffusion, which is required to reach NO production cells and COX cell membrane enzymes. Overall, the inclusion of both lipid nanosystems in the same semisolid base constitutes a promising strategy for autoimmune, inflammatory, and cancerous skin diseases.

## 1. Introduction

Dermatological autoimmune diseases (e.g., psoriasis) [1] are characterized by unbalanced immune responses that contribute to the pathogenesis [2,3]. Both the precise sequence of events and the molecular mediators are yet to be defined, although reported evidence of inflammatory processes involved in these pathogenesis point to the important roles of nitric oxide (NO), a reactive nitrogen species produced by macrophages [4], and cicloxygenase (COX), an enzyme recognized as a mediator of active inflammation at early stages [5,6]. In nonlesional skin conditions, NO is produced in small amounts with an anti-inflammatory effect [7]; however, in patological conditions, large amounts of NO may be produced, leading to the destruction of skin tissues due to the inflammatory response of the immune cells [8]. In general, this type of disease has a major negative impact on patient quality of life due to the characteristics of the symptoms associated with it.

Since there is no effective cure for the different types of chronic inflammatory and autoimmune skin conditions, the main purpose of the available therapeutic options is to improve the quality of life of patients by relieving symptoms and slowing the progression of inflammatory conditions. This highlights the potential use of inhibitors of NO synthesis and COX activity as an effective modality of treatment in autoimmune and inflammatory skin diseases [5,6,8].

Chronic inflammation is also correlated with the development of several cancers, including melanoma [9]. COX-2 is overexpressed in different cancers and is implicated in cancer development and resistance to chemotherapy and radiotherapy treatments [9,10]. COX-2 inhibitors can be used in cancer to reduce the cells’ ability for metastasis development and to sensitize the cancer cells to radio- and chemotherapy [9,10]. The role of NO in melanoma is not yet fully understood and is still controversial [11,12]. Increased NO may be associated with a worse prognosis of the disease [12,13] and inhibitors of NO production are seen by some authors as promising alternatives for melanoma treatment [13].

Several studies have shown that natural bioactives such as resveratrol (RSV–trans-3,4′,5-trihydroxystilbene) and omega 3 (ω_3_) have anti-inflammatory and immunomodulatory effects [14,15,16,17,18,19,20,21]. Indeed, some studies suggest that there are benefits to the use of ω_3_ as complements in the topical treatment of inflammatory and autoimmune skin lesions [19,22], since immunoregulatory actions generally help to reduce the hyperproliferation of keratinocytes [23,24,25]. Aldridge et al. [26] demonstrated that in addition to other anti-inflammatory effects, the ω_3_ lipid emulsion was able to significantly lower NO production involved in many biological functions, such as in the regulation of immune and inflammatory cells [27,28,29].

The anti-inflammatory properties of RSV have been attributed to several mechanisms [20,21,30], including inhibition of COX and inhibition of the NF kB transcription factor, which may reduce the expression of inflammatory genes and the reduction of the intracellular formation of peroxide and superoxide radicals in human skin fibroblasts in vitro. In addition, RSV inhibits tyrosine kinase protein (PTK), which modulates cell proliferation and differentiation, signaling processes in immune system cells, and biological processes involved in inflammatory response and diseases, such as cancer, arteriosclerosis, and psoriasis [31,32,33]. RSV also plays a critical role in regulating the expression of the SIRT1 gene responsible for the activation of Th17 cells, which are important in the modulation of the epidermal keratinocyte proliferation that occurs in some inflammatory skin processes, such as psoriasis [34,35].

There are some problems associated with the use of natural bioactives for the treatment of inflammatory and autoimmune skin diseases; for example, in the case of ω_3_, high susceptibility to lipid peroxidation occurs due to the unsaturated bonds of these fatty acids [36], while for RSV, the cutaneous penetration is limited [37]. A potential approach to overcome low skin permeation is the development of lipid nanosystems, such as liposomes and lipid nanoparticles, which have a lipid composition that is compatible with the fluid lipid matrix of the intercellular route [38,39]. The use of these nanosystems as a local and non-invasive therapeutic strategy is considered a safe and efficient method of treatment for inflammatory and autoimmune skin conditions [1] due to their unique and tunable properties, which promote specific release and absorption characteristics across the stratum corneum, without impairing the functions of the skin barrier, and which improve epidermal, dermal, and transdermal permeation [40]. Various studies have reported the development of liposomes [41,42], as well as nanostructured lipid carriers (NLC) and solid lipid nanoparticles (SLN) [43,44,45], which carry ω_3_ and RSV in order to improve their stability, bioavailability, skin permeation, and biologic activities, such as anti-inflammatory and antioxidant activities, in addition to sustaining the bioactive release and masking unwanted odors and tastes.

In light of our current knowledge, there is no onging study developing formulations for the simultaneous topical administration of these two bioactives. The aim of this study is the rational development and characterization of lipid nanosystems (NLC and large unilamelar liposomes (LUV)) containing fish oils (rich in ω_3_) and RSV for potential use as a topical strategy for autoimmune, inflammatory, and cancerous skin conditions. The uniqueness of this study is also due to the use of two different nanosystems in the same topical formulation, taking advantage of the characteristics of each nanosystem in terms of bioactive delivery. LUV are used to deliver RSV and ω_3_ across the stratum corneum to provide NO and COX inhibitory effects, as well as protective effects against lipid peroxidation. NLC have an occlusive role on the skin surface in order to release ω_3_ at this level, promoting additional cicatrizing effects.

## 2. Materials and Methods

### 2.1. Materials

Omega 3 (ω_3_) capsules were acquired to Myprotein^®^ (England). Resveratrol (RSV), chloroform, and ethanol were purchased from Sigma-Aldrich Química, S.L. (Sintra, Portugal). Precirol^®^ ATO 5 (glyceryl palmitostearate, PREC), Compritol^®^ 808 ATO (glyceryl behenate), Gelucire^®^ 43/01 (glycerol esters of saturated fatty acids esters), Suppocire^®^ D (triglycerides of fatty acid), LabrafacTM Lipophile WL 1349 (medium chain triglycerides), LabrafacTM Lipophile WL (medium chain triglycerides), Maisine^®^ (glyceryl monolinoleate), Peceol^®^ (glyceryl monoleate), and Lauroglycol™ FCC (propylene glycol monolaurate type I) were donated by Gattefossé (Paris, France). Imwitor^®^ 900K (glyceryl monostearate) was supplied by Oxi-Med express, S.A. (Barcelona, Spain), while Dynasan^®^ 114 (trimyristin) and Witepsol^®^ E85 (consisting mostly of triglycerides) were supplied by Sasol GmbH (Hamburg, Germany). 1,2-Dipalmitoyl-*sn*-glycero-3-phosphocholine (DPPC), 1,2-distearoyl-*sn*-glycero-3-phosphocholinephosphatidylcholine (DSPC), and dioleoylphosphatidylethanolamine (DOPE) were purchased from NOF Corporation (Tokyo, Japan). Triethanolamine and Tween^®^ 80 were purchased from Acofarma^®^ (Barcelona, Spain). Gelling PFC^®^ (Carbopol 2001) and glycerin were purchased from Guinama (Valencia, Spain). COX Fluorescent Inhibitor Screening Kit of Cayman Chemical was bought from Bertin Bioreagent (Lisbon, Portugal). Fetal bovine serum (FBS) was purchased from GE Health Care Life Sciences (Logan, UT, USA). Dulbecco’s modified Eagle’s medium/F-12 nutrient mixture (Ham) (DMEM/F-12; 1:1) was purchased from Gibco (Paisley, UK). Dimethyl sulfoxide (DMSO) and phosphoric acid were purchased from Merk (Darmstadt, Germany). Thiazolyl blue tetrazolium bromide (MTT), lipopolysaccharide (LPS), sulfanilamide, naphtylethylenediamide, and gentamicin were purchased from Sigma-Aldrich (St. Louis, MO, USA). All other reagents were acquired from Merck KGaA (Darmstadt, Germany) with p.a. quality and used without further purification.

### 2.2. Methods

#### 2.2.1. Pre-Formulation Studies

In order to evaluate some of the physical and chemical characteristics of the bioactives and lipids under study, pre-formulation studies were carried out for the rational development of formulations, ensuring their stability, effectiveness, and safety. In an initial phase, it is necessary to consider certain factors related to the bioactives and excipients, such as their physicochemical properties and compatibility.

##### Physicochemical Properties of RSV Studied In Silico and In Vitro

To select the most appropriate formulation for topical delivery, different physiochemical and biopharmaceutical properties of RSV were analyzed using SMILES (Simplified Molecular Input Line Entry System) notation and the Chemaxon^®^ software (ChemAxon, Hungary) integrated with the MarvinSketch^®^ module (ChemAxon, Hungary). Several chemical descriptors, such as the ionization, lipophilicity, size, shape, geometry, electrical characteristics, and surface topology, were calculated in silico.

Drug lipophilicity, expressed as logD, was also evaluated in vitro by derivative spectrophotometry using DSPC/DOPE/ω_3_ (6:3:1) large unilamellar liposomes (LUV) prepared using the lipid film hydration method, followed by extrusion [46,47]. For logD determination, samples were prepared containing a fixed concentration of RSV (50 μM) and increasing concentrations of LUV (47–6907 µM). References were identically prepared without the addition of RSV. The control contained only RSV at a concentration of 50 µM, without the addition of liposomes. All preparations were incubated in a thermostatic bath at 37 °C for 30 min. The absorption spectra of samples and the reference were plotted in the 200–400 nm range on a Shimadzu UV/Vis-NIR 3101 PC (Japan) spectrophotometer equipped with a thermostated cell holder at 37.0 ± 0.1 °C. After subtrating the absorption of the reference, derivative spectra were calculated from absorption spectra using a previously published Excel^®^-based routine [48].

##### Screening of Lipidic Excipients

The selection of lipids for the development of lipid nanosystems is a crucial parameter for both stability and performance; therefore, different liquid and solid lipids were selected to evaluate the solubility of RSV in different proportions (1%, 2%, 3%). The solubility was analyzed every 15 min over a total of 60 min at 90°C, checking the presence of crystals of RSV in the molten lipid. The solid lipids tested were PREC, Dynasan^®^ 114, Gelucire^®^ 43/01, Witepsol^®^ E85, Imwitor^®^ 900K, Compritol^®^ 808 ATO, and Suppocire^®^ D. The liquid lipids analyzed were Labrafac^®^ lipophile WL, Labrafac^®^ lipophile WL 1349, Maisine^®^, Peceol^®^, Lauroglycol™ FCC, and Myprotein^®^ fish oil rich in ω_3_.

#### 2.2.2. Formulation Studies

##### Preparation of Lipid Nanosystems

LUV of DSPC/DOPE (6:4) and DSPC/DOPE/ω_3_ (6:3:1); were prepared by classical lipid film hydration method followed by extrusion, as already described [49]. RSV (50 μM) was included in the formulations by direct mixing in the ethanolic solvents prior to lipid film formation. Placebo LUV were prepared in a similar manner by omitting the addition of RSV. 

Since RSV did not solubilize in any of the lipids tested (either liquid or solid), we chose to prepare NLC containing ω_3_ with a double function, i.e., as a constituent of the lipid matrix of NLC and bioactive. In order to avoid the formation of supercooled melts, lipids used to produce lipid nanoparticles should have a melting point higher than 40 °C to remain in solid state at room and body temperatures [50]. The solid lipid selected to develop NLC met this requirement. NLC of PREC/ω_3_ (7:3) were developed by ultrasound technique, as applied by Mendes et al. [51]. The lipid phase was heated at a temperature above the lipid solid melting point on a water bath. The aqueous phase containing surfactant (Tween^®^ 80 2.5% and benzalkonium chloride 0.5%) maintained at the same temperature was added to the lipid phase and homogenized under high-speed stirring, using an Ultra-Turrax^®^ T25 (IKA^®^, Janke & Kunkel GmbH, Staufen im Breisgau, Germany) at 9000 rpm for 5 min, followed by sonication (Bandelin Eletronic UW 2200, Berlin, Germany) at 40% of amplitude for 15 min. The obtained O/W nanoemulsion was immediately transferred to glass vials and cooled down to room temperature in an ice bath in order to originate the NLC.

##### Measurement of Particle Size, Polydispersity Index and Zeta Potential

LUV and NLC were characterized in terms of the mean particle size, polydispersity index (PDI), and zeta potential by dynamic and electrophoretic light scattering (DLS, ELS) using a Zetasizer Nano ZS (Malvern^®^ Instruments Ltd., Grovewood Road, Malvern, Worcestershire, UK).

LUV and NLC were diluted with purified water (*v*:*v*) (1:10 and 1:2, respectively) in order to avoid the multiple scattering effect caused by a high concentration of particles, obtaining the suitable scattering intensity. All experiments were performed under controlled temperature conditions at 25 ± 1 °C on the preparation day (day 0). Size and PDI results were obtained from the correlogram using Zetasizer Nano ZS software (Malvern^®^, Grovewood Road, UK) after cumulant analysis according to ISO 22412:2008 [52]. Zeta potential results were obtained via the conversion of electrophoretic mobility according to the method used by Helmholtz–von Smoluchowski [53].

##### Chemical Composition Analysis

To prove the success of RSV encapsulation, the chemical compositions of LUV with and without RSV were evaluated. The infrared absorption spectra were measured using a Fourier transform infrared spectrophotometer (Spectrum Two™ FT-IR Perkin-Elmer, Waltham, MA, USA) equipped with an attenuated total reflection unit (ATR-FTIR).

Samples were prepared by adding 200 μL of LUV into aluminum crucibles and evaporated to form a lipid film. Measurements were made by pressing the film against the crystal of the ATR accessory in the range of 400–4000 cm^−1^, with a resolution of 4 cm^−1^ and accumulating 64 scans per spectrum. The antioxidant effect of ω_3_ was also evaluated by calculating the ratio of IR bands at 1740 cm^−1^/2960 cm^−1^ from the second derivative FTIR spectra [54].

##### Biophysical Properties Analysis

The microviscosity of LUV was evaluated through determination of the main phase transition temperature (*T_m_*) and the cooperativity (*B*) by dynamic light scattering (DLS) in a Zetasizer Nano ZS (Malvern Instruments, UK) according to the method described by Michel et al. [55]. LUV with and without RSV (1.5 × 10^−4^ M) were incubated at 37 °C for 30 min. The experiments consisted of measuring the hydrodynamic radius and mean count rate (MC, number of detected photons per min) of LUV at several temperatures (5–55 °C). The results were collected as the MCR versus temperature, *T* (in °C), and the data were fitted using the following modified Boltzmann Equation:

(1) $$MCR=b_{1}+m_{1}T+\frac{b_{2}-b_{1}+m_{2}T-m_{1}T}{1+10^{B\cdot \left(\frac{1}{T}-\frac{1}{T_{m}}\right)}}$$

where *m*_1_ and *m*_2_ are the slopes of the linear fits applied to the data before and after the phase transition region, respectively, and *b*_1_ and *b*_2_ are the corresponding y-axis intercepts. Fitting Equation (1) to the experimental data allows one to obtain *B* and *T_m_* of the lipid bilayer from the gel phase to the fluid phase.

In the case of NLC, the phase transition temperature was analyzed by differential scanning calorimetry (DSC) using a DSC 200 F3 Maia^®^ instrument (NETZCH, Bobingen, Germany). Bulk materials (NLC) weighing between 10 and 13 mg were put into sealed aluminum crucibles with perforated caps. As the reference, an equivalent empty crucible and cap were used. The thermal analysis profiles were obtained under a dynamic atmosphere of nitrogen (20 mL/min). For bulk materials, the thermal program involved cooling down to 5 °C (at a rate of 10 °C/min), followed by an isotherm of 8 min and subsequent heating from 5 to 200 °C (at a rate of 5 °C/min). NLC were subjected to the same cooling and isothermal regime as the starting materials, and the heating was carried out from 5 to 90 °C at a rate of 5 °C/min. Data were analyzed using Proteus^®^ 5.2.1. software (NETZCH, Bobingen, Germany).

##### Encapsulating Efficiency

Previously, LUV were diluted with purified water to prevent measurement of erroneous encapsulation efficiency (EE) and drug loading content (DL) values generated by overestimation due to the possibility of drug crystal entrapment between filtered nanosystems. EE and DL were determined indirectly by calculating the amount of non-encapsulated RSV present in the aqueous phase of dispersions and by applying the following Equations:

(2) $$EE(\%)=\frac{{\left[RSV\right]}_{Total}-{\left[RSV\right]}_{Free}}{{\left[RSV\right]}_{Total}}\times 100$$

(3) $$DL(\%)=\frac{{\left[RSV\right]}_{Total}-{\left[RSV\right]}_{Free}}{{\left[Lipid\right]}_{Total}}\times 100$$

Here, 500 μL of RSV-loaded LUV formulations were transferred to Ultracel 50K centrifugal filter devices (Amicon^®^ Ultra, Millipore Corporation, USA) with a 50,000 nominal molecular weight limit pore size. These filter devices were centrifuged (Hettich^®^ Universal 320 centrifuge, Tuttlingen, Germany) at 3000 rpm for 15 min. The amount of encapsulated RSV was determined using a validated UV/Vis spectrophotometry method, using a Shimadzu UV/Vis-NIR 3101 PC (Kyoto, Japan) spectrophotometer, according to International Conference on Harmonization (ICH) Q2(R2) guidelines at the maximum absorption wavelength of the drug (λ_max_ = 305 nm).

##### Incorporation of Lipid Nanosystems in a Hydrogel

Considering the characteristic low viscosity of lipid nanosystems, their incorporation in semisolid forms, such as hydrogels, seems to be an effective strategy for their topical application. The hydrogel was prepared by direct incorporation of lipid nanosystems. Firstly, glycerin was mixed with NLC or LUV. Afterwards, the gelling PFC^®^ (Carbopol-2001, 0.5% (m/m)) was dispersed in this aqueous preparation, which was immediately neutralized with triethanolamine until forming the hydrogel (≈ pH 6.5). The formed hydrogel was left to equilibrate over 24 h at room temperature.

##### Stability Studies

To assess physical stability under storage conditions, lipid nanosystems maintained at refrigerator temperature (4 °C) were analyzed regarding their macroscopic aspect, size, PDI, and ZP at predetermined periods. Analyses were performed in the production day and eight weeks after production. To evaluate the accelerated stability, LUV were subjected to 2 centrifugation cycles at a speed of 3000 rpm for 30 min, then kept at room temperature for 8 weeks, after which the same properties were analyzed.

#### 2.2.3. Evaluation of Pharmaceutic and Therapeutic Performance

##### In Vitro Drug Release Studies

The in vitro RSV release profile from LUV was assessed using the dialysis membrane (Float-A-Lyzer^®^, 3.5 kD, VWR) diffusion technique for 32 h. The release medium was a micellar dispersion (hexadecilphosphocholine) at pH of 5.5 (prepared from a basic solution of sodium decahydrate 0.1 M and an acid solution composed of boric acid 0.02 M and citric acid 0.05 M) to mimic not only the pH of the skin [56], but also the cellular-derived components of skin surface lipids, which consist of phospholipids derived from the corneocyte plasma membrane [38,57]. Lipids are found in the interlamellar regions of the stratum corneum and act as a significant barrier to drug permeation [38]. Briefly, 1.0 mL of LUV was transferred to dialysis membranes and immersed in a container with 6.0 mL of micellar-buffered medium (pH 5.5). The system was kept in a thermostatically controlled water bath mimicking the temperature of the skin (32 ± 2 °C) and under magnetic stirring (200 rpm). At pre-determined time intervals, 1.0 mL of the receptor medium was removed and replaced with the same volume of fresh medium. All experiments were conducted under sink conditions.

The amount of RSV released was measured using a validated UV/Vis spectrophotometer method as previously described. The cumulative RSV released was calculated and expressed as a percentage of the theoretical maximum drug content value.

##### In Vitro Diffusion Studies

The in vitro diffusion of RSV from LUV incorporated into hydrogel bases was evaluated using Franz diffusion cells (V-Series Stirrers for Franz Cells; PermeGear, USA). Polysulfone membranes (Tuffryn^®^ membrane filter with 0.45 µm pore size, PALL Life Sciences, USA) with a diffusion area of 0.64 cm^2^ were fixed between the donor and the receptor compartment and 5 mL of micellar-buffered medium (pH 5.5) was added to the receptor compartment. The membrane was acclimatized at 32 ± 2 °C for 0.5 h prior to the addition of samples to the donor compartment. The receptor liquid was maintained at a constant temperature of 32 ± 2 °C using a circulating water bath and was continually stirred for 24 h. The donor compartment was filled with 400 µL of formulation and aliquots were collected at predetermined time intervals. Each volume removed was and replaced with the same amount of fresh medium. The RSV concentration in the receptor compartment was determined using a validated UV/Vis spectrophotometer method, as previously described.

Diffusion parameters were interpreted from the cumulative amount of release drug per unit membrane area (*Q_R_/A*) versus time (*t*) plot. The steady-state flux (*J_ss_*) and lag time (*t_L_*) correspond to the gradient and x intercept of the linear portion of the plot. *J_ss_* (μg/cm^2^/h) was calculated using the following Equation [58]:

(4) $$J_{SS}=\frac{Q_{R}}{A_{t}}$$

where *t* (h) is the diffusion time, *A* (cm^2^) is the diffusion area, and *Q_R_* (μg) is the diffusion amount of the drug.

The drug permeability coefficient (*K_p_*, in cm/h) was determined using Equation (5):

(5) $$K_{p}=\frac{J_{SS}}{C_{d}}$$

where *C_d_* is the initial concentration in the donor chamber (μg/cm^3^).

##### Occlusive Effect Assessment

Prevention of water loss by lipid nanosystems incorporated into hydrogels was studied by in vitro occlusive test [59]. Here, 50 g of deionized water was added to a beaker, which was covered with a Whatman^®^ cellulose microfibre filter (pore sizes ranged from 0.6 to 0.8 μm) and sealed with Teflon. The tested formulation was evenly spread on the filter surface. The samples were kept at 37 ± 0.5 °C in an incubator for a period of 48 h. The system was weighed at several timepoints to measure the weight loss of water, which is related to the occlusive effect of the formulations.

The occlusive factor (*F*) was calculated according to the following Equation:

(6) $$F=\frac{R-S}{R}\times 100$$

where *R* is the water loss in the control and *S* is the water loss in the system with the sample; that is, *F* = 0 indicates that no occlusive effect occurred and *F* = 100 indicates that the occlusive effect was maximal.

The water vapor transmission rate (*WVTR*) expressed in g/m^2^/day was also calculated following the standard gravimetric method [60]:

(7) $$WVTR=\frac{R-S}{t\times A}$$

where *t* represents the 24 h time point and *A* is the sample testing area (m^2^). Finally, the occlusive effect (%) was calculated by comparison of the WVTR of each lipid nanosystem and the control mimicking the normal transepidermal WVTR of human skin (for healthy human skin, the WVTR is about 5–10 g/m^2^ day) [61,62]:

(8) $$Occlusive\;effect\;(\%)=\frac{WVTR_{CONTROL}-WVTR_{FORMULATION}}{WVTR_{CONTROL}}\times 100$$

##### Rheology Study

After mechanical and thermal equilibration, the rheology analysis was carried out at room temperature using an ST-2001 rotational viscosimeter from J.P. Selecta^®^ (I.C.T, S.L., Abrera, Spain) at a shear rate adequate for the spindle used to obtain the flow properties. Analysis of variance of the shear stress (*Pa*) versus shear rate (rpm) was performed on the production day (*T*_0_) and after two months to evaluate the effects of storage on the stability of the hydrogel formulations.

##### Evaluation of the inhibitory COX and lipid peroxidation effects

The anti-inflammatory effects of lipid nanosystems were evaluated based on their ability to inhibit the cyclooxygenase (COX) activity using a commercial COX Fluorescent Inhibitor Screening Kit (Cayman Chemical Company, Ann Arbor, Michigan in accordance with the manufacturer’s protocol. The reaction between prostaglandin endoperoxide PGG_2_ (resultant from COX activity) and ADHP (10-acetyl-3,7-dihydroxyphenoxazine) produces a highly fluorescent compound named resorufin. The fluorescence of this compound was analyzed with an excitation wavelength of 535 nm and an emission wavelength of 585 nm.

The anti-oxidant activity was evaluated using ATR-FTIR as previously described in Section 2.2.2. The changes in stretching vibration of C=O groups indicate the formation of primary oxidation products such as lipid peroxides. The use of FTIR spectroscopy allows for the direct observation of the appearance or disappearance of bands corresponding to distinct vibrations of functional lipid groups, the changes in which indicate the formation of these primary oxidation products [54]; therefore, the ratio of IR bands at 1740 cm^−1^ (C=O)/2960 cm^−1^ (CH_3_) from the second derivative of the ATR-FTIR spectra was found to be related to the degree of lipid peroxidation (higher ratios indicate higher peroxidation) [54]. Furthermore, to present the results in a quantitative manner, the ratio values were expressed in comparison to the formulation that did not contain any bioactive (DSPC/DOPE) according to the following Equation:

(9) $$Lipid\;peroxidation\;inhibitory\;effect\;(\%)=\frac{IRratio_{DSPC:DOPE}-IRratio_{lipid\_nanosystem}}{IRratio_{DSPC:DOPE}}\times 100$$

##### Evaluation of the effect on NO production

Liposome formulations with and without the bioactives (i.e., ω_3_ and RSV) were evaluated by assessment of their effect on NO production by the murine RAW 264.7 macrophage cell line kindly provided by Maria São José Nascimento from the Laboratory of Microbiology, Biological Sciences Department, Faculty of Pharmacy, University of Porto, Portugal. RAW 264.7 cell culture medium was prepared using DMEM/F12 (1:1) supplemented with fetal bovine serum (10%) and gentamicin (1 μg/mL). Cell cultures were incubated in a 5% CO_2_ humidified atmosphere at 37 °C,. For each assay, cells were seeded at a concentration of 2 × 10^5^ cell/well (96-well culture plate) and incubated for 2 h with the aim of allowing cell adherence. After removal of supernatants, cells were stimulated with lipopolysaccharides (LPS) (1.5 µg/mL) and treated with the desired LUV suspension (1% *v*/*v*) for 24 h [63]. A blank of the compound (compound without cells), a 100% production control (untreated stimulated cells), and a 0% production control (culture media) were added. Supernatants were collected (100 µL), placed in a new 96-well flat-bottom plate, mixed with Griess reagent (100 µL), and prepared by adding equal volumes of 1% *w*/*v* sulfanilamide solution in phosphoric acid (5% *v*/*v*) and naphtylethylenediamide (0.1%) in deionized water. After a 10 min incubation period in the dark at room temperature, the optical density values of the wells were measured (545/630 nm; STAT FAX 3200) and the inhibition of NO production was quantified using the following formula:

(10) $$NO\;production\;inhibition\;(\%)=100-\frac{abs_{lipid\_nanosystem}-abs_{blank}}{abs_{control}-abs_{negative\_control}}\times 100$$

The MTT viability assay was performed to evaluate a possible toxic effect of the liposome formulations against the RAW264.7 murine macrophage cell line. In short, after the removal of supernatants for the Griess assay, the culture medium was discarded and cells were incubated for 4 h with a MTT solution (0.2 mg/mL). After the incubation period, the supernatants were discharged and the MTT formazan product was solubilized with DMSO. Cell viability, determined in relation to the control, was calculated using the following formula:

(11) $$Cell\;viability\;(\%)=\frac{abs_{lipid\_nanosystem}}{abs_{control}}\times 100$$

#### 2.2.4. Statistical Analysis

Statistical analysis was performed on Windows using IBM SPSS Statistics 26. The Kolmogorov–Smirnov was used to validate the normal distribution of the data. One-way ANOVA was performed to test statistical significance in the viability and nitric oxide production experimental assays. Student’s *t* test was applied to explore comparisons. Statistical significance was considered for *p* < 0.05.

## 3. Results, Discussion

### 3.1. Pre-Formulation Studies

For the rational development of pharmaceutical preparations, pre-formulation studies were performed by analyzing the relevant physicochemical characteristics of RSV based on its chemical structure. For this, in silico calculations (Table S1, Supplementary Materials) were used together with several descriptors to understand the most important physiological barriers and the challenges in the development of an effective drug delivery nanocarrier [64].

**Figure 1:**
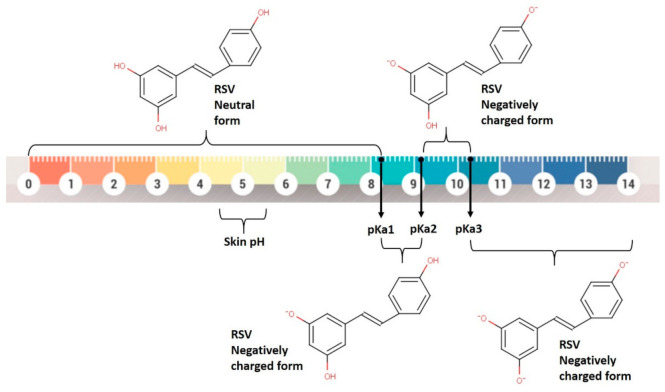
RSV microspecies distribution (neutral and negative) according to pH. The pKa values of RSV are also represented. The chemical structure of RSV was drawn using Marvin Sketch software from Chemaxon^®^.

The most relevant properties in skin permeation are the ionization, solubility, molecular mass, and partition coefficient, as these are the properties that will influence the passage through the stratum corneum [65]. In terms of the RSV ionization profile, the calculated pKa values (Table S1, Supplementary Materials) were close to the experimental values previously reported (i.e., 8.99; 9.63 and 10.64) [66]. At pH values below pKa1, RSV is mostly in its neutral form, while at pH values above pKa1, it is negatively charged (Figure 1); thus, at the physiological pH level of skin (≈ 4–5.5), RSV is in its non-ionized form, which limits its skin permeation.

The aqueous solubility of RSV is extremely low (Figure S1A, Supplementary Materials), with its low concentration at the stratum corneum also compromising its skin permeation [37]. Generally, some properties such as molecular weight < 400–500 Da and 1< logP <3 favor skin permeation [39]. RSV has the required molecular weight (Table S1, Supplementary Materials). At cutaneous pH, the predicted logD and logP values equal 3.4. (Figure S1B, Supplementary Materials), which are close to the reported ones in the literature (logP ~ 3.1) [67], but exceed those required for optimal permeation, which occurs mainly through the interlamellar spaces of the stratum corneum or through the pores of the skin. The logP value also indicates that RSV has moderate to high lipophilicity [68,69], making it appropriate for incorporation into lipid nanosystems (e.g., nanoemulsions, lipid nanoparticles, and liposomes) which have been widely exploited for topical administration of RSV for skin diseases [42,44,70,71,72,73,74,75]. Despite the lipophilicity of RSV, its high PSA value (60.69 Å^2^) indicates that RSV is amphiphilic; therefore, among the lipid nanosystems, liposomes, which have an amphiphilic character and greater fluidity, may be more suitable nanocarriers for RSV encapsulation. SLN and NLC are mostly lipophilic and are less efficient for encapsulating amphiphilic compounds. To confirm that liposomes are appropriate as nanocarriers for RSV, a test was performed using LUV of DSPC/DOPE/ω_3_ (6:3:1) to determine the distribution coefficient (logD) of RSV in the biphasic liposome/water system (Figure S2, Supplementary Materials). The logD_liposome/water_ value of (2.88 ± 0.097) indicates that RSV has a moderate distribution in the LUV but that they are still suitable for RSV encapsulation. The predicted chemical suitability of LUV for RSV encapsulation is consistent with previous reports of lipid-based nanosystems for cutaneous RSV delivery [31,37,76,77,78,79,80].

Additionally, to confirm that NLC may not be suitable for RSV encapsulation, a study was carried out to test its solubility in different solid and liquid lipids, as described in Section 2.2.1. In all tested mixtures, the presence of RSV crystals after 60 min was observed, indicating that RSV is not soluble in the solid and liquid lipids analyzed here; thus, it was not possible to select any of the lipids for the preparation of NLC containing encapsulated RSV and the preparation of NLC was reserved for the inclusion of the other bioactive compound (ω_3_).

In conclusion, the in silico results suggested that encapsulating RSV in lipid nanosystems, particularly liposomes, can improve RSV’s skin permeability. As a result, RSV was only encapsulated in liposomal formulations capable of providing an adequate amphiphilic environment for RSV encapsulation, while ω_3_ was used as both a component of the lipid matrix and a bioactive compound. NLC were ineffective for RSV encapsulation, although they did provide an adequate environment for ω_3_, which was used as a component of the lipid matrix and as a bioactive.

### 3.2. Formulation Studies

**Figure 2:**
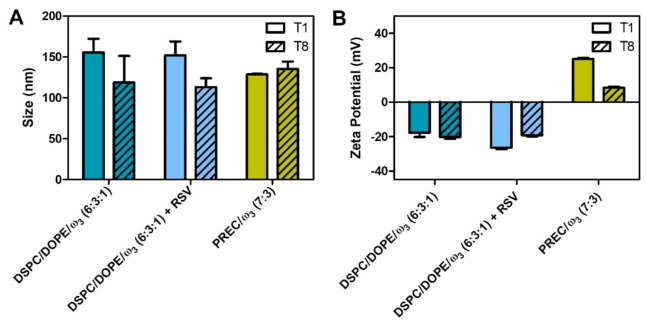
Stability characterization of lipid nanosystems during storage at 4 °C. The data represent the means ± SD of the size (**A**) and surface charge (zeta potential (mV) (**B**) values over 8 weeks.

The physical properties of lipid nanosystems (e.g., size and surface charge) might affect their penetration properties in topical applications. The data in terms of mean size and zeta potential values of LUV prepared with and without RSV; and NLC are presented in Figure 2.

The average size of DSPC/DOPE/ω3 (6:3:1) (156 ± 17 nm) was not significantly different from that obtained by Skibinski et al. (137 ± 12 nm) [41], which also included a fish oil rich in DHA encapsulated in LUV composed of archaelipids. When compared with the LUV obtained by Alaarg et al. (99 ± 16 nm), which constituted ω_3_, 1,2-dipalmitoyl-*sn*-glycero-3-phosphocholine, cholesterol, and PEG_2000_, our lipid nanosystem was significantly larger [81]. On the other hand, DSPC/DOPE/ω3 (6:3:1) containing RSV (152 ± 17 nm) was significantly smaller (*p* < 0.05) than LUV produced by Jøraholmen et al. containing egg-phosphatidylcholine and RSV (206 ± 10 nm) but without fish oil [42]. Despite the differences in the LUV compositions, we also used the extrusion technique, which has been proven to be effective in obtaining sizes close to those reported in the literature.

The average size of PREC/ω3 (7:3) (129 ± 1 nm) was significantly larger (*p* < 0.001) than the one NLC obtained by Shreedar et al. (87 ± 7 nm), which contained ω_3_ and stearic acid, as well as the one obtained by Khan et al. (60 ± 9 nm), which was composed of Compritol^®^ 888 ATO and Capmul MCMC8 [82]. This can be explained by the different components of the lipid matrix as well as the different preparation method (high-pressure homogenization) used by Khan et al. [82].

**Figure 3:**
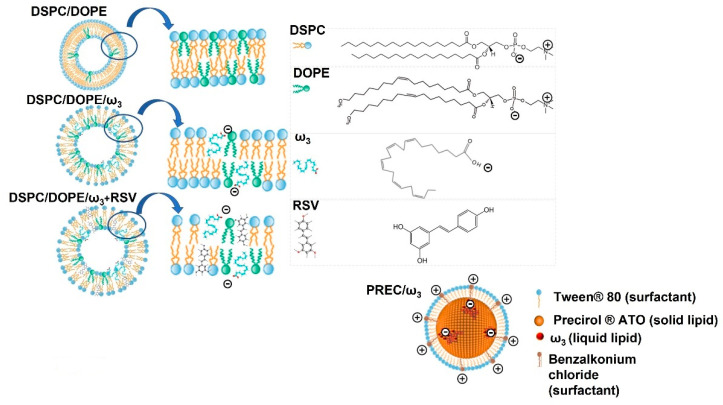
Schematic representation of lipid nanosystems with or without RSV and ω_3_.

Despite LUV and NLC having similar sizes, their PDI values are significantly different (*p* < 0.001), which suggests a different ω_3_ distribution within the lipid matrix. In NLC, ω_3_ is located in reservoirs surrounded by the solid lipid (Figure 3), resulting in lower PDI values (0.198 ± 0.004) than LUV with and without RSV (0.583 ± 0.101 and 0.513 ± 0.011 respectively), where ω_3_ is part of the lipid bilayer, perturbing the lipid packing of the highly ordered lipid DSPC. For comparison purposes, we produced LUV of DSPC/DOPE (6:4) (without ω_3_), which had similar sizes (138 ± 4 nm) but significantly lower PDI values (0.222 ± 0.004, *p* < 0.001).

In summary, the size of both NLC and LUV makes them suitable for topical applications, which require an optimum size range of 100 to 200 nm [83]. Furthermore, small sizes are important when a topical application is intended, because the penetration of a nanocarrier through the stratum corneum decreases as size increases [84]; however, size is a more limiting factor for inorganic nanocarriers due to their structural rigidity. Liposomes are more flexible and moldable, and as a result they penetrate the stratum corneum more effectively [85]. Additionally, the presence of components that disturb lipid packing of LUV (such as ω_3_ and DOPE, known as “edge activators”) enables the formation of nanocarriers with greater flexibility and greater skin penetration [86].

In addition to their size, the surface charge of LUV and NLC was also characterized by measurement of their zeta potential values (Figure 2B). In liposomes without RSV (−17.70 ± 2.46 mV) and with RSV (−26.40 ± 0.90 mV), the presence of a negative surface charge was expected, since ω_3_ acids are fatty acids, whose carboxylic acid group is ionized at pH > 4.5. The significative decrease (*p* < 0.05) of the zeta potential values can be explained by the fact that ω_3_ carboxylic anionic groups are more exposed in the polar headgroup region due to changes in the lipid packing when RSV is encapsulated. NLC contain a cationic surfactant, benzalkonium chloride, at the lipid–water interface, which explains the positive zeta potential (+25.13 ± 0.61 mV).

Over the 8 weeks, there were no significant changes in the average size of the lipid nanosystems (Figure 2A), indicating no aggregation. There were no significant changes in the DSPC/DOPE/ω3 (6:3:1) surface charge in terms of the zeta potential (Figure 2B); however, when RSV is present, its effect on the surface charge is notorious, as indicated by the significant changes of zeta potential values (*p* < 0.0001). This is also related to the abovementioned changes in the polar headgroup region due to RSV distribution in the lipid nanosystem. PREC/ω3 (7:3) also suffered a significant decrease in the surface charge (*p* < 0.0001), most likely due to ω_3_ relocation in the lipid matrix at the interfacial region, which reduced the overall positive charge conferred by the cationic surfactant. Oil relocation in the interfacial region was also observed for NLC in the literature [87]. Despite the decrease in zeta potential, PREC/ω3 (7:3) retains its stability due to the surface coverage by a non-ionic surfactant, which confers steric hindrance and prevents aggregation [88].

**Figure 4:**
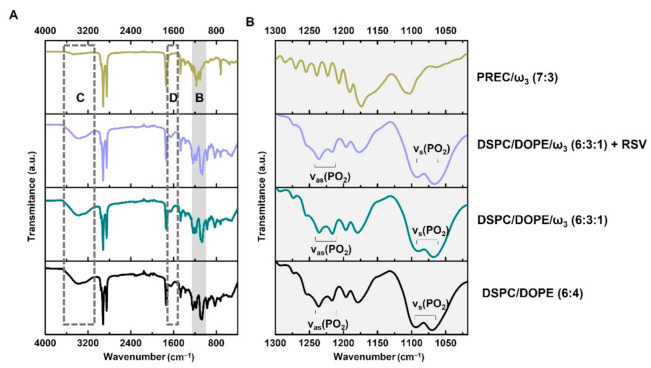
ATR-FTIR spectra of lipid nanosystems with and without ω_3_ and RSV (**A**). The typical vibrations of the chemical groups ν_s_(CH_2_) at 2850 cm^−1^ and ν_as_(CH_2_) at 2916 cm^−1^ are present in all lipid nanosystems. Regions B (vibrations of the phosphate group of liposomes headgroups), C (band ν(N–H) at 3370 cm^−1^ attributed to the choline phospholipids), and D (band δ_as_(N–CH_3_) at 1630 cm^−1^ attributed to the choline phospholipids) are assigned to the regions where NLC and liposomes differ. (**B**) Maximization of phosphate vibrations (Band B).

The chemical characterization of lipid nanosystems was evaluated using ATR-FTIR (Figure 4).

All spectra show the typical lipidic backbone bands: ρ(CH_2_) at 886 cm^−1^; δ_s_(CH_2_) at 1471 cm^−1^; ν_s_(CH_2_) at 2850 cm^−1^; ν_as_(CH_2_) at 2916 cm^−1^. Additionally, other distinct bands are characteristic of LUV but do not appear in the NLC spectra, such as the δ_as_(N–CH_3_) (band D at 1630 cm^−1^) and ν(N–H) (band C at 3370 cm^−1^) of the choline phospholipids. Also present in the headgroup region of the LUV phospholipids are the double simetric (1075–1090 cm^−1^) and assimetric (1221 cm^−1^ and 1200 cm^−1^) vibrations of the phosphate groups, which do not appear in the headgroup regions of NLC lipid components (band B and inset Figure 4B). Comparing the ATR-FTIR spectrum of DSPC/DOPE (6:4) with the spectra of LUV containing ω_3_ or ω_3_ and RSV, it is possible to see that the presence of these bioactives affect the ratio between the assimetric and simetric phosphate bands, confirming their interaction in the headgroup region (Figure 4B). Indeed, the ratio of the asymmetrical stretching band areas (a_1221_/a_1201_) in phosphate can be used to assess the degree of hydration of the headgroup region. An increase of this ratio indicates dehydration around the phosphate groups and possibly the establishment of electrostatic interaction [89]. This was observed in the DSPC/DOPE LUV when ω_3_ was added (the ratio band area value increased from 1.39 to 1.52), most likely because of a stronger interaction between the choline and ethanolamine groups, while the phosphate groups were required to accommodate a fatty acid with a negatively charged polar group. When RSV is added to DSPC/DOPE/ω3, the ratio between band areas decreases slightly to 1.50, indicating the formation of hydrogen bridges between the RSV and the polar heads of the LUV phospholipids.

The biophysical and thermodynamic properties of the lipid nanosystems were also characterized (Figure S3, Supplementary Materials). The DSPC/DOPE/ω3 (6:3:1) showed two transition temperatures T_m1_=30.14 ± 0.14 °C and T_m2_=41.72 ± 0.56 °C, indicating the coexistence of two lipid phases, one more fluid and less cooperative phase (*B* = 223.77 ± 21.73) rich in DOPE and ω_3_, and another ordered and cooperative phase (*B* = 1170.62 ± 42.73) corresponding to DSPC-enriched domains (Figure S3A). When RSV was encapsulated in the formulations, the integrity of LUV was maintained, although both lipid phases were more ordered, as proven by the increases of the transition temperatures to T_m1_=33.73 ± 0.14 °C and T_m2_=53.24 ± 3.95 °C (Figure S3A). This ws in agreement with the reported biophysical effect of RSV in lipid membranes [90]. After RSV introduction, the cooperativity levels of the two lipid phases were similar (*B* = 595.13 ± 108.44 and 452.00 ± 72.11), indicating a homogeneous distribution of RSV in both phases. The PREC/ω3 (7:3) has a melting temperature T_m_ = 60 °C, which is slightly lower than the T_m_ of PREC (62 °C) and still ensures that NLC are solid at room and body temperature (Figure S3B).

The RSV entrapment efficiency for DSPC/DOPE/ω3 (6:3:1) was 71.76 ± 9.23%, which was not statistically different from the results obtained by Jøraholmen et al. [42](80 ± 4%) and Caddeo et al. [75] (73.1 ± 3.3% and 79.7 ± 3.8%); however, if the DL is considered, RSV loading in our formulation was significantly higher (14.35 ± 1.85 %, *p* < 0.0001) than the values obtained by Caddeo et al. (3.68 ± 0.17% and 2.72 ± 0.13%).

### 3.3. Potential Pharmaceutic and Therapeutic Performance

**Figure 5:**
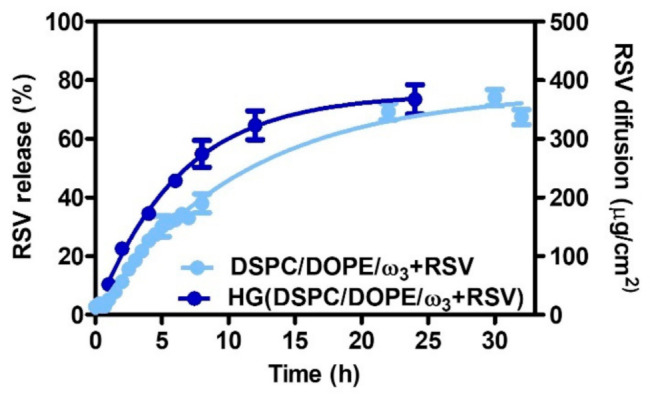
Release and diffusion profile of RSV from DSPC/DOPE/ω3 (6:3:1) in free form and included in the hydrogel (HG). The data represent the means ± SD.

Several parameters can be used to predict the pharmaceutic and therapeutic potential of topical cutaneous formulations in vitro. Firstly, the release profile of RSV from DSPC/DOPE/ω3 (6:3:1) was evaluated (Figure 5).

LUV released RSV in a sustained way following a first-order kinetic profile, where a maximum of 75.96 ± 1.90% was released over 30 h and with a kinetic constant of 𝑘 = 0.09276 ± 0.004556 h^−1^. A similar RSV release after 8 h (≈ 40%) was reported by Jøraholmen et al. [42]. The nonlinear fitting with other mathematical models (zero-order, Higuchi, and Korsmeyer–Peppas) was also tested and is presented in Table S2 (Supplementary Materials).

Besides analysing the release of RSV from the lipid formulation, it is important to assess whether the planned final semisolid formulation maintains the RSV diffusion capacity. For this purpose, Franz cell models using artificial membranes have been described as important characterisation tools for the evaluation of the performance of semi-solid pharmaceutical products for topical administration [91]. Regardless of the importance of these assays, any interpretation should be cautious because the membranes used are artificial and do not completely mimic the complex diffusion phenomena observed in vivo; however, the stiffness, mechanical resistance, thermal stability, and oxidation resistance of polysulfone membrane filters mean they are widely used in diffusion studies [88] and their use to assess the topical drug delivery is recognized by regulatory entities such as the FDA [89]. As such, RSV release from LUV included in a hydrogel, HG(DSPC/DOPE/ω3), was assessed by quantification of the RSV diffused through the polysulfone membranes in Franz cells (Figure 5).

The diffusion flux (*J_ss_*) of RSV across the membrane barrier was 22.21 ± 0.35 µg/cm^2^/h and the permeability (*K_p_*) of the RSV through the diffusion membrane was 0.0254 ± 0.0004 cm/h. Using the logD_liposome/water_ calculated (Figure S2, Supplementary Materials) using the algorithm created by Potts and Guy [92], and knowing the saturation concentration of RSV (3 mg/100 cm^3^), the theoretical value of the RSV permeability coefficient was calculated as 0.0269 cm/h, which was very close to the experimental values achieved (0.0254 ± 0.0004 cm/h).

**Figure 6:**
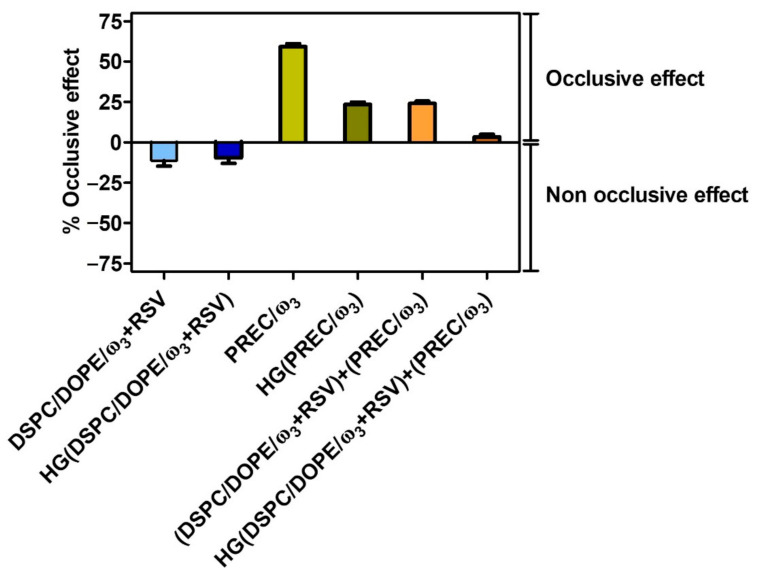
Occlusive effects (%) of free lipid nanosystems and of those included in the hydrogel (HG). The occlusive effects were calculated according to Equation (8). The data represent the means ± SD.

In addition to drug release from lipid nanocarriers and diffusion from the lipid nanocarriers incorporated in the semisolid base, it is also important to understand whether the formulations can prevent water loss from the skin, i.e., can have an occlusive effect that can interfere with the normal transepidermal water loss (WVTR) of human skin [61,62]. This occlusive effect could be beneficial in increasing the hydration of the stratum corneum, and consequently could influence the percutaneous absorption and the efficacy of bioactives [93]. For this, the occlusive effects of LUV, NLC, LUV+NLC, and their HG incorporated counterparts were compared (Figure 6).

There was no occlusive effect of LUV, whether free or incorporated into the HG base. This lack of occlusive effect does not favor skin permeation [94], which is not a positive result. Indeed, some permeation is required because the pathogenesis of inflammatory processes in some skin lesions involves not only epidermal skin structures, but also deeper and more vascularized dermal regions; however, this result, which would not be so favorable for topical application in healthy skin, is not discouraging for the intended application, i.e., for damaged skin (such as psoriasis), which is more permeable [95]. Furthermore, because of their flexible structures, liposomes may permeate the skin via other mechanisms than occlusion [96]. The NLC produced the highest occlusive effect (59.31 ± 1.79% decrease of WVTR, Figure 6), which points out the ability of these lipid nanosystems to reside on the skin surface, which can be beneficial in increasing the hydration of the stratum corneum, producing an emollient effect on the skin surface and consequently influencing the percutaneous absorption of liposomes [93].

Hydrogels incorporating the two lipid nanosystems presented significantly smaller (*p* < 0.0001) occlusive effects, which were mostly due to the high water content of the HG base; however, the presence of NLC in the HG still counteracts the lack of occlusive effects observed for LUV. These data seem to underline the interest in using a semisolid base containing both lipid nanosystems.

**Figure 7:**
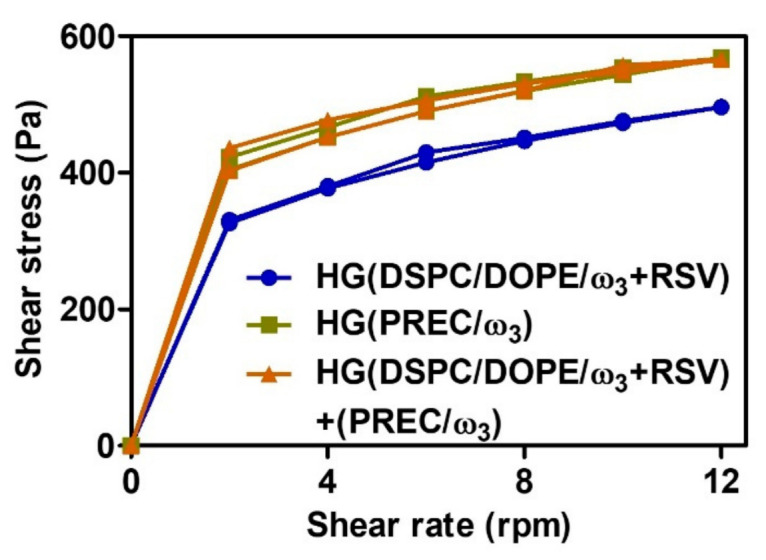
Rheograms of lipid nanosystems included in the hydrogel (HG). The data represent the means ± SD.

After incorporation of the lipid nanosystems in the HG, rheological analyses were performed (Figure 7).

The rheograms demonstrated that all HG exhibited a typical flow curve of non-Newtonian pseudoplastic behavior, which was visible from the viscosity decrease with the increasing shear rate, which caused the breakdown of the hydrogel network structure (reduced by the gradual decrease of viscosity). These semisolid bases do not present thixotropy; that is, there is no decrease in viscosity with time, since the ascending and descending curves of the rheograms are practically overlaped and without the characteristic hysteresis of thixotropic behavior [97]. This means that after cessation of high shear stress, HGs can rapidly recover their consistency and elastic properties, promoting their retention. For the topical administration of semisolid bases, the pseudoplastic behavior is interesting, favoring the application of the base while the patient makes movements to spread the product, since during these movements its viscosity decreases, then when the movements stop, the viscosity returns to its initial values [97,98]. When both lipid nanosystems were incorporated into the HG base, the viscosity increased when compared to the HG that only contained LUV. This is consistent with the role of NLCs in compensating for the lack of occlusive effects of LUVs when both lipid nanosystems were incorporated into the HG.

The potential therapeutic activity of the lipid nanosystems was initially screened regarding their inhibitory COX effects and the effects against lipid peroxidation (Table 1).

DSPC/DOPE/ω_3_ (6:3:1)+RSV and DSPC/DOPE (6:4)+RSV demonstrated similar COX inhibition effects, although these effects were significantly higher than all other lipid nanosystems (Table 1, *p* < 0.0001), free RSV (91.84 ± 0.50%, *p* < 0.0001; data not included in Table 1) or free ω_3_ (92.90 ± 0.51%, *p* < 0.001; data not included in Table 1), or the COX inhibitor, DuP-697 (supplied by Cayman Chemical Company) (83.19 ± 0.46%, *p* < 0.0001; data not included in Table 1). Lipid nanosystems without RSV presented significantly lower COX inhibition effects (*p* < 0.01), which were not influenced by the presence of ω_3_ (Table 1); therefore, the presence of RSV in the lipid nanosystems seems to be required to achieve higher anti-inflammatory effects via COX inhibition. In the NLC, the COX inhibitory effect was about 75.99 ± 0.42%, which was lower than free ω_3_ (92.90 ± 0.51%, *p* < 0.001; data not included in Table 1), probably because in NLC ω_3_ is an integral part of the lipid matrix, being inside the nanoparticle structure and not fully exposed to the surfaces of nanoparticles, as is the case for free ω_3_. The same can be concluded when comparing DSPC/DOPE/ω_3_ (6:3:1) to free ω_3._ It should be emphasized, however, that the differences in COX inhibitory effects of ω_3_ between NLC and LUV could simply be attributable to the larger concentration of ω_3_ in NLC. Although free RSV and free ω_3_ had strong COX inhibitory effects, their incorporation in lipid nanosystems is required for in vivo activity, because COX is bound to to the membranes of fibroblasts and its inhibition requires bioactives to pass through the skin layers to the dermis.

LUV have significantly higher lipid peroxidation inhibitory effects (*p* < 0.05) than NLC, except in the case of LUV containing both RSV and ω_3_, which present similar effects to NLC (Table 1). These results seem to indicate that each bioactive has an anti-oxidant effect, although the presence of both bioactives is not synergic.

Since LUV containing the bioactives demonstrated higher COX inhibitory effects, their potential anti-inflammatory effects by NO production using LPS-stimulated RAW264.7 murine macrophages were evaluated (*p* > 0.05) (Table 2).

All formulations inhibited NO production, although no statistical significance was observed between them. Although the NO inhibition effect observed for LUV without bioactives (i.e., ω_3_ or RSV) was not statistically significant (ANOVA analysis), the *p*-value was nearly significant (*p* = 0.063). With this in mind, and merely for comparison purposes, Student’s *t* test was performed to explore differences between independent DSPC/DOPE/ω_3_ (6:3:1) and DSPC/DOPE (6:4) samples. A statistical difference was observed (*p* < 0.05). The effect of the LUV formulation on NO detection was not due to a toxic effect against RAW264.7 macrophages, as inferred by the viability of stimulated macrophages treated with the different formulations in relation to the control. The observation that a higher inhibitory effect of NO production occurs only when ω_3_ is included in LUV without RSV suggests a higher contribution of ω_3_ at this level and a lack of synergic effects between the two bioactives. Since the anti-inflammatory effect of RSV is well documented in the literature [99], our results point to an anti-inflammatory mechanism via COX inhibition rather than interference with NO production.

## 4. Conclusions

In conclusion, since the success of topical administration is highly dependent on the type of carrier, the proposed use of LUV and NLC provides a combined strategy in order to overcome the skin delivery limitations of the bioctives. On one hand, it is expected that NLC remain at the skin surface, promoting an increase of the occlusive effect, blocking the diffusional water loss, and increasing the hydration of the stratum corneum. This occlusive mechanism has positive effects in terms of bioactive permeation and in reduction of the typical exfoliative skin lesions. On the other hand, LUV containing edge activator components are more flexible and are expected to reach deeper skin layers. LUV are expected to provide permeation enhancement of RSV, similar to other lipid-based nanosystems reported for cutaneous RSV delivery, which facilitate skin permeation and provide controlled RSV release at the desired sites of action [31,37,76,77,78,79,80] (which in this case were the dermis sites where inflammation mediators are overexpressed). Although expected, the different skin locations of NLC and LUV should be confirmed through microscopic observations using in vitro or ex vivo split-thickness skin models.

While in vivo studies are still needed to confirm the in vitro studies results, the above discussed effects of lipid nanosystems carrying ω_3_ and RSV bioactives on inhibition of COX activity, lipid peroxidation, and NO production give us an indication of their anti-inflammatory and anti-oxidant effects, making their topical application a promising strategy for autoimmune, inflammatory, and cancerous skin diseases.

## Figures and Tables

**Table 1:** Inhibitory effects of Cox and lipid peroxidation of the lipid nanosystems. The data represent the means ± SD.

Lipid Nanosystem	COX Inhibition (%) ^1^	Lipid Peroxidation Inhibition (%) ^2^
DSPC/DOPE (6:4)	13.98 ± 0.08	0
DSPC/DOPE (6:4) + RSV	95.42 ± 0.52	85.61 ± 4.28
DSPC/DOPE/ω3 (6:3:1)	46.24 ± 0.25	84.83 ± 4.24
DSPC/DOPE/ω3 (6:3:1) + RSV	94.61 ± 0.52	82.34 ± 4.12
PREC/ω3 (7:3)	75.99 ± 0.42	72.50 ± 3.62

Note: ^1^ values were obtained using a commercial COX inhibitor fluorescence screening kit using COX as a positive control; ^2^ values were obtained using Equation (9); values represent the means ± SD (*n* = 3).

**Table 2:** Effects of liposome formulations on nitric oxide (NO) production by RAW264.7 macrophages stimulated with lipopolysaccharide (LPS).

Lipid Nanosystem	NO Production Inhibition (% of Control)	Cell Viability (% of Control)
DSPC/DOPE (6:4)	21.60 ± 4.61	102.53 ± 6.22
DSPC/DOPE (6:4) + RSV	31.37 ± 4.72	97.50 ± 3.08
DSPC/DOPE/ω_3_ (6:3:1)	39.77 ± 4.80	95.52 ± 1.98
DSPC/DOPE/ω_3_ (6:3:1) + RSV	30.47 ± 1.69	98.48 ± 2.24

Values represent the means ± SEM (*n* = 4).

## Data Availability

Data sharing not applicable.

## Supplementary Materials

The following are available online at https://www.mdpi.com/article/10.3390/pharmaceutics13081202/s1: Table S1: In silico prediction of several physicochemical descriptors of RSV. Figure S1: In silico prediction of: (A) aqueous solubility of RSV as a function of pH; (B) logD of RSV as a function of pH. Figure S2: Schematic representation of the distribution coefficient of RSV (logD) in the biphasic liposome–water system determined by derivative spectrophotometry. Figure S3: Main phase transition temperature (T_m1_, T_m2_ or T_m_) in LUV (determined by dynamic light scattering) and in NLC (determined by differential scanning calorimetry). Reference [49] is cted in Figure S2.
