# Supplementary material for: Omega-3- and Resveratrol-Loaded Lipid Nanosystems for Potential Use as Topical Formulations in Autoimmune, Inflammatory, and Cancerous Skin Diseases
Source: Pharmaceutics. 2021 Aug 4;13(8):1202. doi: 10.3390/pharmaceutics13081202 (PMC8401194; doi:10.3390/pharmaceutics13081202)
Supplement: Supplementary file 1 [file pharmaceutics-13-01202-s001.zip › pharmaceutics-1306972-supplementary.pdf]

pharmaceutics

Supplementary Materials: Omega-3- and Resveratrol-Loaded Lipid Nanosystems for Potential Use as Topical Formulations in Autoimmune, Inflammatory, and Cancerous Skin Diseases

Ana R. Caldas, José Catita, Raul Machado, Artur Ribeiro, Fátima Cerqueira, Bruno Horta, Rui Medeiros, Marlene Lúcio and Carla M. Lopes

Table S1. In silico prediction of several physicochemical descriptors obtained from resveratrol (RSV) chemical structure using Chemaxon® software (version 5.3.1.).

Mw (g.mol^{-1})	logP	Aqueous solubility	PSA (Å^2)	VWSA (Å^3)	pKa1	pKa2	pKa3	H Donors	H Acceptors
228.5	3.4	Low	60.69	308.38	8.49	9.13	10.14	3	3

[1] Abbreviations: logP - $\log_{10}$ of the octanol/water partition coefficient; Mw – molecular weight; pKa - negative $\log_{10}$ of the ionization constant; PSA - polar surface area; VWSA - Van der Waals surface area

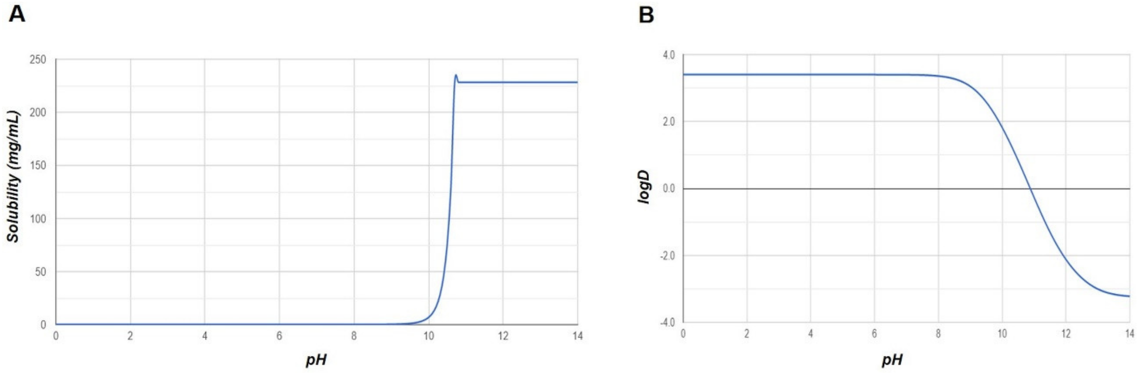

Figure S1. In silico prediction of: **(A)** Aqueous solubility of RSV as a function of pH; **(B)** logD of RSV as a function of pH. Both predictions were made with Chemaxon® software.

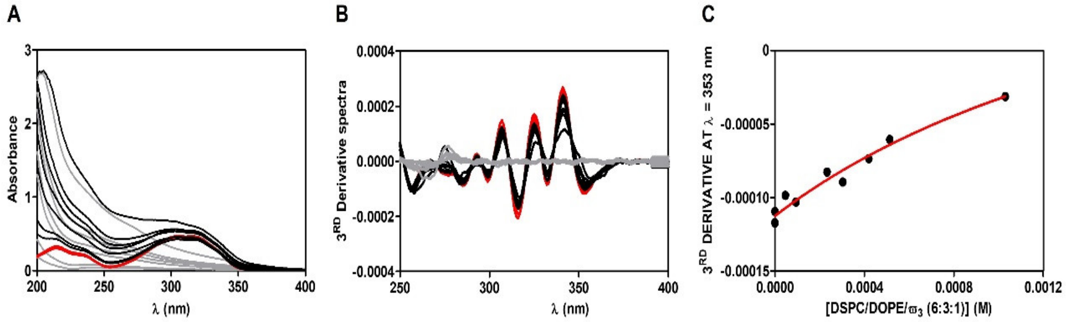

Figure S2. Schematic representation of distribution coefficient of RSV (logD) in the biphasic liposome/water system determination by derivative spectrophotometry: **(A)** RSV (50 μM) absorption spectra in aqueous buffered phase pH = 5.5 (red spectrum), after incubation with increasing concentrations of LUV (47– 6907 μM) prepared in the same conditions (black spectra) and increasing concentrations of LUV (47– 6907 μM) in the absence of RSV (grey spectra); **(B)** Resultant spectra after LUV references subtraction; **(C)** Non-linear fitting of derivative absorbance at λ = 353 nm as a function of LUV concentration was as previously described [49].

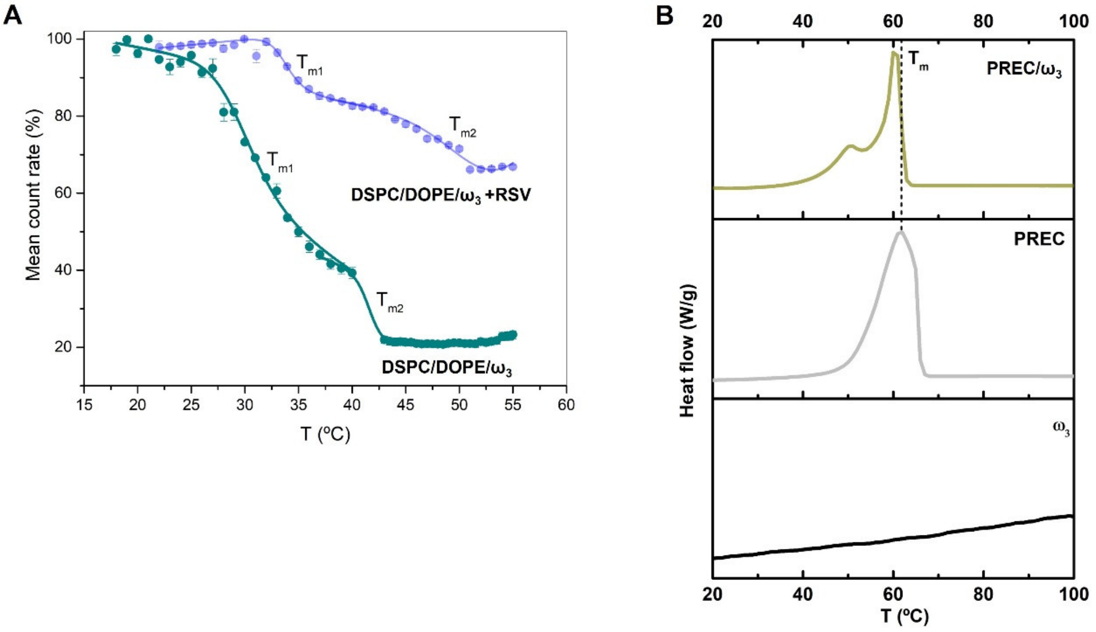

Figure S3. Main phase transition temperature (T_{m1}, T_{m2} or T_m) in LUV (A – determined by dynamic light scattering) and in NLC (B – determined by differential scanning calorimetry).

Table S2. – Fittings of RSV release kinetics from DSPC/DOPE/ ω3 (7:3:1) liposomes in buffered micellar medium using mathematical models.

1st Order Kinetics	Parameters	R^2	R^2 adjusted
$F_{max}(1-e^{-kt})$	F_{max}= 75,96 ± 1,897<br>k = 0,09276±0,004556	0,99912	0,98643
**Korsmeyer–Peppas model**			
at^n	a = 10,67±0,8910<br>n = 0,5688±0,02928	0,9649	0,96743
**Weibull model**			
$F_{max}\left(1-e^{(-at^b)}\right)$	a = 0,08758 ± 0,008031<br>F_{max}= 74,27 ± 2,580<br>b = 1,053 ± 0,06931	0,9915	0,96851
**Higuchi model**			
$kt^{0,5}$	k =12,76 ± 0,3904	0,9535	0,99805

a is a constant of geometric and structural incorporation that considers the pharmaceutical form; n is a release parameter that represents the drug diffusion mechanism, being based on Fick's law, (a value of n equal to or less than 0.5 indicates a Fickian diffusion, for values between 0.5 and 1 it indicates a non-Fickian transport. *K* is the Higuchi dissolution constant.

References

49. Fernandes, E.; Soares, T.B.; Gonçalves, H.; Lúcio, M. Spectroscopic Studies as a Toolbox for Biophysical and Chemical Characterization of Lipid-Based Nanotherapeutics. *Front. Chem.* **2018**, *6*, doi:10.3389/fchem.2018.00323.
